# Supplementary material for: Nonverbal communication in selfies posted on Instagram: Another look at the effect of gender on vertical camera angle
Source: PLoS One. 2020 Sep 11;15(9):e0238588. doi: 10.1371/journal.pone.0238588 (PMC7485807; doi:10.1371/journal.pone.0238588)
Supplement: Attachment — Submitted filename: Review_PONE-D-20-08908.pdf [file pone.0238588.s001.pdf]

The authors analyze selfies posted on Instagram and test whether there are sex differences in camera angle. Camera angle can alter various features of photographs, including the perceived facial appearance of targets. Thus, different camera angles may be employed to emphasize or enhance certain characteristics. A previous study found that on Tinder, men were more likely to post selfies from a low camera angle, whereas women were more likely to post selfies from a high camera angle. In the current study, the authors test whether these findings replicate on a social networking (rather than a dating) website. The study provides novel insights into how men and women differently utilize camera angle when taking selfies. Yet, there are several issues that should be addressed.

Major issues:

1) I would like to see a more detailed explanation of the communicative intentions underlying different camera positions. Which objective features of photos are altered by different vertical camera positions? Which inferences do these differences trigger in perceivers (e.g., Hehman et al., 2013)? And finally, what are the ultimate explanations for sex differences in this practice? In other words, the theoretical depth of the paper could be improved.
2) Whereas the general research questions are explicitly stated at the end of the introduction, the specific hypotheses for the current study were less clear to me. It is true that Instagram is generally used for different purposes than Tinder. However, it is unclear what predictions regarding sex differences in camera positioning would follow from this observation. One the one hand, the effect might only emerge in online dating environments where looking attractive is very important. On the other hand, people might aim to enhance their perceived attractiveness in many situations. Especially on social networking sites like Instagram or Facebook, people might be concerned with looking attractive. These considerations should be discussed. There might also be empirical work on (a) the motives underlying activity on Instagram and (b) how widespread the motive to look attractive is.
3) Most importantly, the main independent variable of interest needs to be specified more clearly. Three features need to be disentangled: camera position, head orientation, and gaze. Did the authors focus only on selfies in which targets looked directly into the camera and heads were vertically angled to face the camera? In this case, camera position would not change the appearance of the face itself. Only the background might change with a higher (lower) camera position leading to more visibility of stimuli below (above) the face. Or did head orientation and gaze also vary? Crucially, this changes the appearance of the face and leads to different trait attributions. For example, people can manipulate their perceived facial width-to-height ratio, which influences various trait attributions (Hehman et al., 2013). This could be achieved (a) by tilting the head up or down while gazing directly into a camera at eye-level (Hehman et al., 2013) or (b) by gazing directly into a camera below eye-level without tilting the head. In short, camera position by itself only changes one, arguably less interesting, aspect of selfies (what is visible in the background). Differences in facial appearance (and resulting inferences), which seem to be the main focus of the current paper, are a function of camera position, head orientation, and gaze.

Minor issues:

4) "It has been proposed (Bruno, Uccelli, Pisu, Belluardo & De Stefani, 2020) that these signals may be conceptualized, within the two dimensional framework of the picture, as analogous to the non-verbal signals that are provided by spatial behaviours in three-dimensional interactions between human as well as non-human animals (Hall, 1966; Hediger,1955; von Uexküll, 1957)" (p.3). This sentence could be clearer, especially as it seems to introduce the theoretical framework of the study. Are the authors proposing that we should study communicative intentions of selfies by focusing on image qualities that can be objectively extracted from the photo's two-dimensional structure (e.g., face-to-body ratio)? What would be the advantage of this focus compared to competing approaches?
5) Who coded the gender of targets? Were there multiple raters? What was their level of agreement?
6) Tables 3 and 4 could easily be combined in one table.
7) In the General Discussion, the authors compare their results with previous results from Tinder. However, some direct comparisons with Sedgewick's study, such as "female selfies are much more likely to display a frontal angle on *Tinder* than they are on *Instagram*" (p. 8), are not supported with statistical tests. It seems like the authors have the data needed to test for differences between their findings and the findings of the Sedgewick study. These results should be presented in the Results section and not the GD.
8) "Our assessment of interindividual consistency further corroborates this conclusion, suggesting that female and males are similarly consistent in their choices." (p. 8) Where is this result reported? It looks like the authors only report sex differences in consistent choices of low camera angles, but not sex differences in overall consistency.
9) "In a communicative context that is relatively unspecific, therefore, they choose the more expressive camera angles from above more than they do neutral frontal angles." In a context explicitly emphasizing mate selection, such as the dating site *Tinder*, female choices of neutral angles instead become more frequent in comparison to cameras above and below, possibly as a consequence of Western social norms that disapprove advertising sexual availability more in women than men "" (p. 9) The argument was not clear to me. First, why would the same sexual signal be seen as inappropriate in a mate choice context, but as appropriate in what the authors call a neutral context? If anything, should sexual signaling be seen as less appropriate in a non-mate choice situation? Second, appearing more attractive is different from signaling sexual availability. Is there evidence that camera position influences perceptions of the latter? Finally, even though Tinder and Instagram are designed for different purposes (dating vs. socializing) people might be strongly concerned with their attractiveness in both contexts. Attractiveness may be a strong driver of popularity on Instagram. That is, the comparison between Instagram and Tinder is not a clean comparison of contexts that are characterized by strong vs. weak attractiveness motives. This limitation of the current study should be made explicit.

Bastian Jaeger

References

Hehman, E., Leitner, J. B., & Gaertner, S. L. (2013). Enhancing static facial features increases intimidation. *Journal of Experimental Social Psychology*, *49*(4), 747–754. https://doi.org/10.1016/j.jesp.2013.02.015
